# Navigating end-of-life care dilemmas: a qualitative inquiry of nurses’ and midwives’ knowledge of euthanasia and circumstantial factors influencing euthanasia in a resource-constraint setting

**DOI:** 10.1186/s12912-024-02527-2

**Published:** 2024-11-26

**Authors:** Hadiru Iddris Mumuni, Merri Iddrisu, Luke Laari, Gladys Dzansi, Lydia Aziato

**Affiliations:** 1 Department of Nursing, School of Nursing and Midwifery, University of Health and Allied Sciences, Ho-Volta Region, Ho, Volta Region, Ghana; 2 Department of Adult Health, School of Nursing and Midwifery, College of Health Sciences, University of Ghana, Accra, Ghana; 3 Department of Public Health, School of Nursing and Midwifery, College of Health Sciences, University of Ghana, Accra, Ghana; 4 University of Health and Allied Sciences, Ho, Volta Region, Ghana

**Keywords:** Euthanasia, Ghana, Health facilities, Midwives, Nurses

## Abstract

**Background:**

Deaths can be caused by terminal illnesses, accidents, or natural disasters. However, medically, death can be hastened by healthcare providers, patients themselves, or their relatives. In advanced cancers where the patient’s quality of life is compromised, Euthanasia can be used to hasten death. Inadequate medical resources and low socioeconomic status have been cited as factors influencing Euthanasia. This study sought to explore nurses’ and midwives’ knowledge and perspectives on Euthanasia in resource-constrained health facilities.

**Methods:**

A qualitative exploratory, descriptive design was used to recruit 24 nurses and midwives from three major referral hospitals, two regional hospitals, and one district hospital. Data was collected through individual in-depth face-to-face and telephone interviews. Braun and Clarke’s (2006) thematic data analysis approach was used.

**Findings:**

: Three main themes and eight subthemes were generated from the data: the main themes include Knowledge of Euthanasia, health system resource constraint-driven euthanasia and family resource constraint-motivated euthanasia. The findings indicate that nurses and midwives lack understanding of some terminologies related to the act. Strict policy decisions, inadequate resources, and misunderstanding of palliative care resulted in the practice of Euthanasia. Additionally, unbearable pains and financial constraints of families of critically ill patients made them request Euthanasia in the health facilities.

**Conclusion:**

The study highlights the challenges of end-of-life care in resource-constrained settings, emphasizing the need for provider training, increased healthcare capacity, and clearer national guidelines for ethical decision-making.

**Supplementary Information:**

The online version contains supplementary material available at 10.1186/s12912-024-02527-2.

## Introduction

In resource-limited countries such as Ghana, healthcare systems face significant challenges due to the overwhelming burden of diseases such as malaria, tuberculosis, and HIV/AIDS, compounded by the growing prevalence of non-communicable diseases [1]. In response to the aging population’s needs, health sector stakeholders advocate establishing hospice centers and extending palliative care services. However, the debate surrounding end-of-life care is complex. Some argue that it is more humane to allow individuals to die with dignity rather than endure prolonged suffering, a perspective influenced by cultural attitudes toward death and burial practices in African societies [2, 3].

Euthanasia is a controversial subject in most countries worldwide due to different ethical-legal as well as socio-cultural views. There are now laws in the Netherlands and Belgium permitting euthanasia, having brought it into common practice years ago (2002 under strict conditions of unbearable suffering for the Dutch). Moreover, its broader criteria and special provisions for minors have triggered a debate about maturity and consent in Belgium. Medical assistance in dying (MAID) became legal across Canada in 2016, with a legislative focus on “clear safeguards to protect the eligible people. Apart from this, there is ongoing debate to increase the eligibility criterion for mental health problems [4].

In the U.S., rules differ state by state, with some allowing it and others banning assisted suicide. As a result, contentious discussions on patient rights vs. healthcare responsibilities abound. The debate over euthanasia weighs respect for individual autonomy against dangers to especially vulnerable communities and is, therefore, an essential issue in medical ethics today [2, 4, 5].

While euthanasia remains illegal in Ghana, some healthcare workers, including nurses and doctors, engage in forms of passive euthanasia, often motivated by socioeconomic factors [6]. Approximately seven countries in the developed world have legalized euthanasia, and while some healthcare professionals in Ghana oppose the practice due to ethical concerns, others find moral justifications for their involvement in certain circumstances [7, 8].

Nurses in the West who practice euthanasia often base their decisions on ethical principles, including respect for autonomy, beneficence, non-maleficence, and justice [9, 10]. A qualitative study in Ghana indicated that extreme poverty, inadequate medical training, and a lack of life-saving medical equipment significantly influence the occurrence of passive euthanasia [6, 11]. Furthermore, public opinion in Ghana shows a 22% acceptance rate for euthanasia and a call for its legalization [8].

As the world becomes increasingly interconnected, low-resourced countries like Ghana may be influenced by Western practices, potentially leading to the acceptance of euthanasia to alleviate the pressures on healthcare systems, particularly in acute and chronic care settings. This study, therefore, aims to explore the knowledge and perspectives of nurses and midwives regarding the practice of euthanasia in resource-constrained health facilities, highlighting their experiences with patients in severe pain.

## Methodology

### Study design

We used a descriptive exploratory qualitative design to explore the views of Nurses and Midwives on the practice of Euthanasia in advanced cancer care. The design was deemed fit because little is known about the phenomenon under study (Euthanasia) in this context [12].

### Study setting

This study was conducted among nurses and midwives in cancer care in six major health facilities in Ghana’s coastal, forest, and savanna zones. The sixteen administrative regions of Ghana were divided or clustered into three geographic/ecological zones (northern or Savanna, Middle or Forest, and southern or Coastal) to ensure that every area would be represented in the sample. The north and middle zones have five regions each, while the south has six.

### Study population

The researchers were interested in qualified nurses and midwives working in healthcare facilities where cancer care services are rendered in Ghana.

### Inclusion and exclusion criteria

 Nurses and midwives working in chronic wards in hospitals (municipal, regional, and teaching) where cancer patients are admitted receiving care were the targets for recruitment. Student nurses, Rotation nurses, and nurses working in chemotherapy centers were exempted from the study.

### Sample size and sampling technique

We used purposive and Snowball sampling methods to select 24 participants based on information power [13] and the concept of saturation [14]. As this study aimed to recruit nurses and midwives with experience caring for cancer patients, snowball sampling was particularly appropriate. These groups, though obviously not ‘hidden’, are centrally enrolled (i.e., they have a self-selected or quasi-voluntary referral process) and are less likely to be available for study by conventional sampling methods, being busy with clinical work. We recruited a few purposively selected participants early in the study, and they were used to identify additional participants who met specific inclusion criteria using snowball sampling. This strategy not only meant that we could reach a wider variety of professionals but also diversified the range of experts whose experiences and knowledge about euthanasia were to be included. This makes snowball sampling the ideal choice to allow a full exploration of potential issues surrounding health care in Ghana. The concept of information power states that fewer participants are required to obtain enough depth of comprehension if each participant possesses more knowledge pertinent to the phenomenon under study [13]. We ensured that participants in this study had the necessary knowledge to contribute to the research by concentrating on nurses and midwives with substantial experiences in caring for cancer patients. The idea of saturation further influenced our sample size. Data saturation occurs when no new themes or information can be obtained from the data set. We evaluated saturation during the data collection process by reviewing the data and noting instances in which more interviews produced repeated information. Through this iterative process, we concluded that 24 participants were enough to adequately represent the target population’s range of experiences and viewpoints.

### Data collection procedure and tool

We developed a semi-structured interview guide for collecting data for this study. A semi-structured interview guide was developed and aligned with the study objectives. Similarly, the development of the guide was conducted in an iterative way based on a literature review and expert consultations with palliative care experts to ensure that all important questions were included [14]. Following this, in order to fine-tune the guide, we piloted it with four nurses. This pilot was conducted as a test to help us evaluate the clarity and flow of questions, but also re-write some parts based on participant feedback. (The guiding questions are available as a supplementary document).” Some of the questions asked include: *Please tell me what you know about Euthanasia (Probe: Source of information*,* during training*,* during practice*,* on the internet); Please share with me the types of Euthanasia you know of (Probe: Active*,* Passive*,* Indirect*,* Indirect*,* Involuntary;* Please tell me what the laws in Ghana say about Euthanasia. (Criminal, Legal); Can you please share with me your thoughts on Euthanasia in health facilities? *Please share with me the type of Euthanasia you have seen being carried out of practice in any health facility you have worked in before.*

Before data collection started, nurses and midwives who work in the wards or units where cancer patients are cared for in the institutions targeted for the study were contacted to help recruit suitable participants. The purpose of the study was explained to them, and they helped recruit nurses and midwives who satisfied the inclusion criteria. When prospective participants were identified, we verified their eligibility to ensure they provided care to cancer patients in the wards and were not student or rotation nurses and midwives. Afterward, the participants were informed of the study’s objectives so they could understand them. Arrangements were made with participants who agreed to participate in the survey regarding the convenience of place and time for either face-to-face or telephone interviews to be scheduled. Before every interview, participants were asked to consent, either verbally or in writing, depending on the interview mode. Participants were told they had a right to withdraw from the study anytime they wished to do so without any consequences. All interviews were audio recorded with the participant’s consent. The interview lasted between 30 and 45 min.

### Data analysis

Data analysis was done alongside data collection. The interviews were transcribed verbatim and analyzed using Braun and Clarke’s six-phase guide for the thematic analysis [15]. Data were analyzed both inductively and deductively.

- Familiarization with the Data: Two researchers independently read the transcripts multiple times to gain an in-depth understanding of the data. This initial phase allowed them to immerse themselves in the content and identify preliminary insights.
- Generating Initial Codes: The researchers systematically coded the data by identifying significant features relevant to the research questions. Each transcript was broken down into manageable segments, and codes were assigned to capture the essence of the data.
- Searching for Themes: Codes that reflected similar ideas were grouped to form subthemes. This phase involved organizing the data into broader categories that captured the underlying patterns and meanings.
- Reviewing Themes: Three authors reviewed the generated themes and sub-themes. During this group review process, the authors discussed the themes to establish consensus. This process helped guarantee that the generated themes and subthemes reflected well in data content and were coherent with research objectives, thereby improving the quality and trustworthiness of the analysis.
- Defining and Naming Themes: Once the themes were finalized, the researchers clearly defined and named each theme to ensure they effectively communicated the essence of the findings.
- Producing the Report: The final phase involved integrating the thematic analysis into the broader context of the study, allowing for a coherent presentation of the findings.

### Rigor

Guba and Lincoln initially proposed the four criteria for ensuring trustworthiness in qualitative research [16] and [17] modified. These criteria guided the researchers, including credibility, transferability, dependability, and confirmability. Participants were engaged for a long time (30 to 45 min) to be able to explore the phenomenon entirely. During data collection, the researchers reechoed what participants said to be sure they were correctly quoted. After data collection, participants were consulted to ensure data accuracy by sending transcripts to those who opted to read through their transcript, its interpretation, and analysis (member-checking). The researchers gave vivid descriptions of how data was obtained to maintain its dependability. In addition, an audit trail regarding study processes is maintained to ensure confirmability. A thick contextual description of the entire study is done, and participants’ accounts were kept when presenting the findings to ensure transferability.

### Ethical consideration

Noguchi Memorial Institute for Medical Research Review Board, Ghana, approved the study ethically (NMIMR-IRB CPN 034/21–22). The study was explained to participants in simple language, including their rights to opt-out at any point they feel uncomfortable continuing. Before data collection began, each participant provided informed consent. The participants were assured of confidentiality through anonymity and the elimination of any possible identifiers, such as the real names of their institutions and unit of work which were omitted in the demographic characteristics.

## Findings

### Participants’ demographic characteristics

The researchers recruited 24 nurses and midwives, four males and 40 females, from six health facilities. The rank of the nurses ranged from senior staff nurses and midwives to the position of Principal Nursing/Midwifery Officer. The years of experience of these professionals is from three to 16 years. Two nurses had a specialty in oncology, two specialized in palliative care, four in midwifery, and the remaining in general practice. Four of the participants were Muslims, one was a Buddhist, and 19 of them were Christians. Details of the participants’ characteristics are found in Table 1 below.

**Table 1:** Participants demographic characteristics

Participants Identity	Age	Gender	Profession	Grade	Year of Practice	Participants’ Highest Educational Level	Religious denomination
NM1	33 yrs.	Female	General Nurse (GN)	Senior Nursing Officer (SNO)	5yrs.	BSc	Christian
NM2	29yrs.	Male	G N	Senior Staff Nurse (SSN)	3yrs.	BSc Nursing + Membership	Christian
NM3	37yrs.	Female	Oncology	Nurse Specialist	8yrs.	Diploma in Midwifery	Muslim
NM4	32 yrs.	Female	GN	SNO	4yrs.	BSc Nursing	Christian
NM5	31yrs,	Female	Midwife	Midwife Officer	4yrs.	Diploma in Nursing	Christian
NM6	36yrs.	Female	GN	SNO	7yrs.	BSc Nursing	Christian
NM7	46yrs.	Female	GN	Principal Nursing Officer (PNO)	16yrs.	Diploma in Nursing	Christian
NM8	35 yrs.	Female	Midwife	Principal Midwifery Officer	7yrs.	MPhil Nursing	Christian
NM9	29 yrs.	Male	GN	Staff Nurse	3yrs.	Diploma in Nursing	Muslim
NM10	31yrs.	Divorced	Midwife	Nursing Officer (NO)	6yrs.	Diploma in Nursing	Christian
NM11	42yrs.	Single	GN	Midwifery Officer	10yrs.	BSc Midwifery	Christian
NM12	36yrs.	Female	Palliative Care Nurse	Nurse Specialist	9yrs.	Diploma in Midwifery	Christian
NM13	42 year.	Male	GN	NO	13 yrs.	BSc in Nursing	Christian
NM14	36yrs.	Male	GN	NO	8yrs.	BSc Midwifery	Christian
NM15	34 yrs.	Female	GN	Staff Midwife	6yrs.	Diploma In Midwifery	Christian
NM16	35yrs.	Female	Palliative Care Nurse	Nurse Specialist	10yrs.	Master’s in public health + Specialization	Christian
NM17	28 yrs.	Female	GN	NO	3yrs.	BSc Nursing	Muslim
NM18	36 Years	Male	GN	SNO	7yrs.	Diploma in Nursing	Christian
NM19	37	Female	Midwife	SMO	6yrs.	Diploma in Midwifery	Christian
NM20	40	Female	GN	PNO	12yrs.	Diploma in Midwifery	Christian
NM21	443	Female	Oncology Nurse	Nurse Specialist	14yrs.	BSc Nursing	Christian
NM22	38	Female	GN	SNO	7yrs.	BSC Nursing	Christian
NM23	36	Female	GN	SNO	8yrs.	BSc Nursing	Buddhist
NM24	32	Male	GN	NO	3yrs.	BSc Nursing	Muslim

**Source: Interview Data**

### Themes and sub-themes

The data generated three main themes and eight subthemes: Knowledge of Euthanasia, healthcare system resource constraint-driven Euthanasia, and family Resource constraint-motivated euthanasia. Table 2 below details the themes and their subthemes.

**Table 2:** Themes and sub-themes

No.	THEMES	SUB-THEMES
1.	Knowledge on Euthanasia	a. Inadequate Knowledge of the Concepts of Euthanasia b. Knowledge of the Legal Position of Euthanasia
2.	Health System Resource Constrained-Driven Euthanasia	a. Strict Policy Decisions b. Inadequate Medical Resources c. Misunderstanding of Palliative Care
3.	Family Resource Constrained Motivated Euthanasia	a. Verbally Requested Euthanasia b. Action Determined Euthanasia c. Economic Situation Prescribed Euthanasia

Source: Interview Data

### Knowledge of nurses on euthanasia

This theme specifies the nurses’ understanding of Euthanasia. Here, participants defined and explained the varied ways they understand Euthanasia, including the types available. The interaction on their knowledge of Euthanasia brought about two subthemes, which are explained with participants’ verbatim quotations below.

#### Inadequate knowledge of the concepts of euthanasia

Though participants had some understanding of what Euthanasia means, they were deficient in understanding in details what the types mean and who is responsible for undertaking the act. As some conceived euthanasia as intentional killing, others thought of it as mercy killing.

The participants believe that Euthanasia is undertaken to ease the suffering of a patient through death. However, some of their narration justified that some of them were deficient in processes that led to the execution of the act and not the sole decision of the healthcare professional.

*“Euthanasia*,* in simple terms*,* is mercy killing where you think that a patient is suffering too much and you want to end it for the patient peacefully or quietly using drugs”* NM7.

“*It’s all about mercy killing. The people who are suffering so much are in a lot of pain*,* and you*,* like*,* sometimes the pain is just too much for the patient to keep bearing for so long. That’s what you do*,* you give them some drugs or something to make them die to stop the suffering”* NM4.

Some participants could mention some of the types of Euthanasia and explain them. However, some were unsure of their explanations, although they narrated situations that suggested passive and active Euthanasia. The following quotations are what participants shared about their understanding of the types of Euthanasia.

*“I don’t know whether there are forms*,* but I know that sometimes they either inject you with something or when you are on a ventilator*,* they take you off. The person can state that when I get to some stage*,* you can kill me*,* or you*,* the person taking care of the patient*,* can kill the patient”.* NM9.

“*There can be two forms*,* active and passive; the active form is the one you intentionally inject the drug*,* and the passive one is taking a patient off a ventilation or disconnecting the patient from oxygen source.”* NM11.

#### Knowledge of the legal position of euthanasia

When it comes to the legal position of Euthanasia in Ghana, some of the participants knew that the constitution of the country frowns at Euthanasia and that there are consequences for persons engaging in the act. Some participants gave specifications about the punitive measures that can be taken against a person who engages in the act. Whether the act is requested by the patient or initiated by a professional, the act remains illegal, and the consequences may include a jail term for the one performing this act.

*“the law does not support or make Euthanasia legal*,* neither does it even support suicide. Suppose the patient tells you to give them drugs to take under your watch once you do that. You are an ally who has attempted suicide*,* so you can go to jail for that. So the law criminalizes Euthanasia in this country”.* NM10.

*“I know that patients who are having this terminal disease that maybe they have been on life support*,* and then when it comes to a time*,* they need to take them off the life support for them to go away peacefully. This can happen in other countries but not here because it is illegal in our country”* NM15.

The theme found that participants had limited knowledge about legal and ethical issues of euthanasia but were generally knowledgeable about basic aspects. The lack of standard information led to poorly founded assumptions and potential implications for patient care. The study emphasizes the need for improved education on euthanasia and end-of-life care.

### Health system resource constrained-driven euthanasia

Constraint-driven Euthanasia emerged to explain structural issues within the health system that facilitated the occurrence of Euthanasia. Participants described reasons Euthanasia of any form is undertaken in multiple healthcare facilities. These professionals interviewed attributed the occurrence of Euthanasia in some health facilities to inadequate or lack of basic and advanced resources to assist in health care. In some cases, Euthanasia occurs due to policy decisions some health facilities adopted and also a misunderstanding of the palliative care concept.

#### Strict policy decisions

Strict adherence to policy decisions is important in every institution. However, participants narrated situations where policies in some health facilities constrained nurses and midwives from operating within the scope and mandate of their profession to save lives.

Some specific hospital policies deterred nurses and midwives from giving patients the needed care because if they did the consequences were victimization from superiors. Participants shared that in some hospitals when a staff member takes medications from the pharmacy for a patient without payment for some time, the money is taken from the nurse/midwife. This act prevented the nurses and midwives from going for medications for patients in the absence of their relatives.

*“I cannot go begging for drugs or go taking drugs to come and give to all clients when I know that at the end of the day if the client does not pay*,* in my facility*,* it will be taken from my salary because when you are taking the drug for a patient without money*,* you have to write your name so if the patient does not pay You will have to pay so I cannot go taking medications for all the patients when I know I also have myself and family to feed.”* MN 14.

*“Sometimes you feel like helping patients*,* but when you consider the aftermath of you doing what is good for the patient*,* I quickly withdraw my thoughts. In my units*,* you are supposed to replace all medications used within 24 hours for others to use*,* and if you fail*,* your name is sent to the nurse manager. What follows is unpalatable*,* so I would rather keep their medications in the cupboard and have my peace than use it on a patient”.* NM 8.

The participants narrated situations that led to the unfortunate death of patients as they were not able to pay the outstanding medication bills and could not afford additional medications for days. However, the system is aware that they reported a debilitating condition.

“*I was once on night duty*,* I went to the pharmacy to take a patient’s medications because relatives were not around*,* and I needed to administer some. The pharmacist demanded payment for the first medications in three days before giving them to others. At this point*,* the patient had no medications*,* and we were told not to come for any medications again*,* so we had no choice but to leave the patient there*,* and in a few days*,* he expired. His condition was bad*,* so I think the family just wanted a death certificate and mortuary to keep him after death”.* NM 20.

#### Inadequate medical resources

The inadequacy of the needed supply of medical consumables and non-consumable medical resources, as well as the behavior of personnel, contributed to the practice of involuntary Euthanasia in health facilities. The healthcare providers who, in this case, were the participants of this study, passively performed Euthanasia to preserve the few resources they had for people they thought should not die because of their health situation at the expense of the vulnerable.

*“… So*,* one afternoon*,* I went*,* and I could see that he had become weak and was gasping for breath. Even though the face mask was worn to give Oxygen*,* the saturation was not good because the cylinder was barely empty. I stood there watching*,* and as a junior staff*,* I couldn’t move on to other patients. The man was looking at me and gasping till he finally gave up. That day*,* I couldn’t do anything again”.* NM19.

Participants who were midwives equally shared instances where Euthanasia was practiced in the labor wards and the babies’ units in various hospitals where they work. For them, they withdraw essential services like Oxygen to allow most asphyxiated babies to die after several attempts of resuscitation with no promising results.

*“It happens in the babies’ units often. You have delivered a baby with severe asphyxia*,* and this baby is not doing well; you need to give oxygen continuously and check the baby often to see if the condition is improving. If the condition is still the same*,* for how long are you going to give Oxygen to this child? We know how Oxygen is difficult to come by*,* so at times*,* we discontinue it to see what will happen and through this act we lose most of them or discontinue it for them to go so that we can use it for others who will survive”* NM16.

In other instances, the inadequate supply of significant resources like ventilators in the neonatal intensive care unit makes staff make certain decisions at the expense of the lives of babies when they are faced with challenging situations. The non-availability of adequate ventilators in some neonatal intensive care units in some hospitals influenced the practice of Euthanasia among babies.

*“The one I witnessed was where I had my rotation some years ago*,* and I don’t want to mention the name. And it was a baby*,* a preterm baby*,* and a macrosomic baby*,* so all through*,* the babies were on ventilators. Then*,* that hospital could not keep the babies on the ventilators for long because others had to be on the ventilators*,* too. After all*,* the hospital did not have enough. They had to decide to take the babies off the ventilators*,* and they could not make it”.* NM9.

*“I witnessed one in the babies’ unit of my hospital; it happened that we had no ventilator in the NICU*,* so when the babies are born*,* and the Apgar score is bad*,* we are asked to keep the babies in a cot somewhere so that they can just pass on gently*,* it is sad but what can you do? The things are not there to work with”.* NM5.

#### Misunderstanding of palliative care

In some instances, nurses and midwives poorly understood the concept of palliative care, so they denied seriously ill patients some life-saving care because they were declared to receive palliative care. In facilities where physicians indicated that patients receive palliative care, the patients were instead denied essential care and left to die. The following quotations from participants justify the various acts of Misunderstanding of palliative care healthcare professionals displayed.

*“I have observed that the moment the tag ‘palliative care’ is given to a patient*,* all other care ceases*,* right from vital signs checking to serving of medication. When you get to the patient*,* time is running out. Just jump to the next patient. So*,* there is no bed bath at times because common items for the bath are not there*,* and there are also not many*,* so nothing much is done for the patient*,* and he will just be lying down. We get such cases and bypass them; the sad aspect is that they eventually die”.* NM15.

“*There was this woman who was one of our top priority patients. We would go around her a lot of the time to assess her*,* but then the very time that the doctors came and wrote palliative care in her folder*,* the next day*,* I went to the older woman*,* and my senior colleagues whispered to me*,* hey*,* she’s on palliative care. So*,* that means a gradual withdrawal of care has started. I’ve seen it done to kids*,* older adults*,* and everything else. So once it is written in the patient’s document as receiving palliative care*,* it means we are technically going to practice euthanasia”.* NM13.

According to the participants’ narrations, most of the Euthanasia occurring is seen in patients with advanced cancers where there is the realization that the patients have no more years to live.

*“the first time I experienced the act was when we cared for a patient with colorectal cancer*,* and the doctors came on rounds and wrote palliative care for the patient. Subsequently*,* I realized that when we are on rounds and get to the patient’s bedside*,* we don’t attend to him. We do not do any checks*,* examinations*,* or follow-ups on him. I was off duty for two days*,* and when I resumed*,* we were told that the patient would die soon*,* so we should not waste our time on him. We were told to focus our attention on other patients”.* NM8.

Though involuntary Euthanasia is being practiced in some health facilities, other unsuccessful attempts at Euthanasia happened due to ethical conflicts that ensued among the staff. This is where staff who are asked to abandon patients to die look within themselves and refuse to go by specific orders and continue to give care.

*“We had a patient who was to go for prostatectomy*,* and the urologist called us to prepare the patient and said that he was on his way to the hospital. We sent this patient to the theatre*,* and the anesthetist said that the patient was gone. Can’t we see that his legs are not moving again? Meanwhile*,* the patient was alive*,* and he said we should disconnect the gadgets to save resources for other patients. Even the blood transfusion he was receiving*,* he commanded us to stop it. He refused to give the anesthesia*,* so the surgery was not done. When the Oxygen in the cylinder finished*,* he told us not to change the cylinder. My conscience did not allow me to do that*,* so I went for another one to set it up for him. I continued giving the blood*,* and after my off duty*,* I came to meet the patient alive. So*,* from my point of view*,* I think what the anesthetist was trying to do is Euthanasia*,* withdrawal of care”.* NM15.

This theme revealed that end-of-life decision-making is influenced by structural aspects of healthcare, including lack of resources, policies, and misunderstandings about palliative care. Addressing these systemic issues can improve patient outcomes and align with ethical guidelines.

### Family resource constrained motivated euthanasia

The request for Euthanasia from family members for their loved ones was mainly made not as a result of their wanting the person but rather because of unbearable pain experienced by the patient and lack of money to buy medications and other essential items to continue with care make their request and/or portray actions for their patients to die peacefully. The act is either verbally requested or through their actions in the form of neglect and refusal to purchase live-sustaining medications or pay for them to be on gadgets.

#### Verbally requested euthanasia

Here, families orally request healthcare professionals to do something or give the patient a medication that could help to end the patient’s life. Some of the participants’ own families have ever requested Euthanasia for their chronically ill patients. In some instances, patients’ children and close family members devise ways of convincing healthcare professionals to hasten the death of their loved ones.

*“The doctors prescribe expensive medications for patients to reduce the pain that was unbearable irrespective of the families’ financial status. In the case of my grandmother*,* who suffered cervical cancer*,* we were drained financially*,* so it got to a time we stopped buying the medications*,* and he died because we could not continue to buy them; we were suffering too much”.* NM 17.

*“There was an instance where relatives of a patient who was terminally ill on the ward for a very long time came to the ward and kept asking if we do not have anything to give to the man for him to go*,* especially his daughter*,* and at the last minute she was like as for my father he has taken a concoction and for that matter*,* unless we take him home he will never die here without we performing a ritual so we the health workers should aid his death for them.* NM2.

#### Action determined euthanasia

In addition to relatives verbally requesting Euthanasia for their sick relatives, they also portrayed specific actions that indicated that they wanted their relative to die through neglect or withdrawal of treatment. In some, it is either the family neglects the patient entirely at the hospital, or they will refuse to buy medication with the excuse that they have no money.

*“Some family members will come to dump their relatives at the hospital and go for about a month*,* but they will never come there to see the patients. Whether the clients are on medications or not*,* they do not care. They neglect him to die out of pain*,* hunger*,* loneliness*,* and sadness. However*,* you*,* the nurse*,* can only do it up to a point and leave it. You know we do not have control over the medications in the pharmacy. We sometimes buy food for them*,* but how long will you continue to do that? You can only do up to a point*,* but you can’t say you will buy all the medication for the person*,* so we stop at a point*,* and nature acts on them; that is what happens”* NM 15.

While some relatives come to dump their relations at the hospitals without buying medications or food for them, others tell the nurses and midwives that they do not have money to continue care, so their relatives should be discharged for them to go home. At other times, the family, together with the patient, will request for them to be discharged home for lack of money, which eventually leads to the death of the patient. The nurses and midwives sometimes assist in buying the medications and other required items but cannot use all their salaries the patients as they also have families to care for.

*“In some of the major cases we handle on the ward*,* a relative will come and will be like I don’t have enough money; my money is finished*,* so discharge the person for us to go home and sometimes*,* the patients also support the relatives to be allowed to go home because they can’t pay the bill. Mostly*,* when they say that*,* they write a letter to that effect*,* and when you refuse them*,* they leave the patient unattended*,* and as a nurse*,* there is little you can do so later the patient dies*,* and they will come*,* and you will direct them to the mortuary*,* it happens a lot*,* very sad”.* NM23.

“*I once cared for a man who was very ill*,* and though we didn’t do what the relative wanted us to do*,* they stopped buying medications because it got to a time they knew the man would not survive*,* and we could not use all our salaries to buy the medication continuously*,* so we withdrew treatment*,* and he finally died.”* NM14.

#### Economic situation prescribed euthanasia

Apart from some health facility policies that restricted nurses and midwives from giving care and saving lives coupled with misunderstanding of palliative care, some participants confirmed that the economic situation of some patients and the withdrawal of care led to the act of Euthanasia.

*“As for Euthanasia*,* it is happening on the ward. Nobody is giving anybody injections to kill anybody*,* but your economic situation and withdrawal of care determine your fate.* NM1.

*“I have witnessed a lot of indirect Euthanasia on the ward*,* and I saw a man with liver cancer die because he was not economically sound. In the beginning*,* the patient was strong and not seriously ill. He did not have insurance*,* and as time went on*,* no relative came around to pay him or purchase medications. We ceased treatment*,* and I said to myself*,* if this man were to have money*,* he could have been revived*,* and I thought we were wicked for not even giving the needed care even though he had no money”.* NM19.

Despite some family members’ cohesion and their intentional neglect of patients, a participant shared a view that most of the time, some relatives will beg for care to be rendered to their loved ones on credit and not request Euthanasia. In Ghana, cultural beliefs and values often place a strong emphasis on preserving life and maintaining family bonds, which could influence attitudes toward Euthanasia. The participants believe euthanasia requests are among some selected few family members and not among the majority.

*“I will say that it happens about 0.5% of the time*,* in the sense that in most of the cases I have seen*,* even when they do not have financial support*,* they beg to be treated on credit for them*,* but they don’t go like we don’t have money so kill the person. Mostly because they want the person to get better*,* they will persuade you to give care for payment later”.* NM10.

*“I do not think our culture encourages such practices*,* and you know*,* at times*,* families will go the extra mile to find money to care for their loved ones. Some people will come to the Church for financial support*,* and the Church will support them. Scarcely will you hear that a family has abandoned their own at the hospital.”* NM22.

The theme highlighted the intricate influence of cost pressures on end-of-life decision making. Many times, euthanasia was requested by family members not because their loved one should die but to avoid suffering that cannot be alleviated or prevented, and they cannot bear the truth about what care costs. This highlights the desperate need for readily available specialist palliative care services and resources that help end-of-life patients, which in turn can put financial pressure on families by providing top-quality care, ultimately decreasing their “in favor” of euthanasia biases.

## Discussion

Euthanasia, the purposeful killing of a patient to ease their distress is without a doubt one of the most controversial and contentious issues globally [18, 19]. It is a complex subject with bioethical, legal, and social aspects that have triggered more than one debate around the role of health professionals, but above all nurses and midwives, in its implementation [18, 20]. The controversial nature of euthanasia practice has gained attention in recent years as global health trends create unique ethical challenges for healthcare providers [20]. This study sought to explore nurses’ and midwives’ knowledge and perspectives on the practice of euthanasia in resource-constrained health facilities. After engaging 24 experienced nurses and midwives through face-to-face and telephone interviews, it became clear that euthanasia is practiced in many health facilities for various reasons. The main themes generated from the data were awareness about euthanasia, the restricted power of health system resources on practicing it as well as the influence of family resource restrictions on deciding on euthanasia.

In settings where resources are scarce, misunderstandings and systemic pressures have a profound influence on attitudes around euthanasia. High demand for palliative care exists in many countries, but because the healthcare systems are overburdened and the quality of available care unsatisfactory, euthanasia is resorted to even though both skills and readiness to provide the best care may be lacking. Moreover, the limitations on family resources, both emotional strain and financial pressure, seldom lead to calls for euthanasia being expressed verbally.

We need a wider discussion here about euthanasia that includes education, health policy, and even better palliative care to tackle these problems. Our goal in this discussion is to clarify the implications of our findings and provide recommendations from the ground for filling gaps and alleviating complications relevant to euthanasia and end-of-life care.

### Knowledge of euthanasia

Nurses and midwives play a critical role in end-of-life care and decision-making, making their knowledge and attitudes toward euthanasia significant [21, 22; 20]. Although participants demonstrated some understanding of euthanasia, they lacked detailed knowledge about its types and the responsibilities of those involved. Previous research indicated that many nurses and midwives have limited knowledge of euthanasia due to its illegal status in Africa [2]. Furthermore, studies indicate that awareness and understanding vary widely across countries, with healthcare professionals in jurisdictions where euthanasia is legalized generally possessing a better grasp of its ethical implications [6].

In Ghana, where euthanasia remains illegal, healthcare professionals often lack formal guidance on this issue. This knowledge gap leads to ethical dilemmas for nurses and midwives when faced with terminally ill patients seeking relief from suffering. The absence of a legal framework hinders effective decision-making, potentially resulting in suboptimal care outcomes for patients who could benefit from more informed discussions about end-of-life options [8, 11]. Consequently, the inability to effectively address patients’ end-of-life preferences hinders the delivery of compassionate and appropriate care, ultimately impacting the quality of life for those they serve.

Understanding the global landscape of euthanasia and its legal status is crucial for healthcare providers in Ghana. While some participants recognized the constitutional prohibition against euthanasia, their limited understanding of the legal nuances may lead to confusion in patient care discussions. This lack of clarity can complicate the ethical landscape, as nurses and midwives may feel unprepared to navigate the moral implications of their actions while respecting patient autonomy [4, 5, 10].

### Health system resource constrained-driven euthanasia

Globally, healthcare systems vary significantly in resource allocation, which influences the ethical and practical considerations surrounding euthanasia. In developed countries, discussions about euthanasia often focus on principles of autonomy and patient choice facilitated by abundant palliative care services [5]. In contrast, Ghana faces substantial challenges in healthcare resource allocation, which can directly impact patient care.

This study found that euthanasia practices in Ghanaian health facilities often arise from systemic issues such as inadequate funding, a shortage of healthcare professionals, lack of effective pain medications and insufficient infrastructure. When access to pain relief, hospice care, or life-sustaining treatments is limited, patients and their families may perceive euthanasia as their only viable option to alleviate suffering. It is crucial to emphasize that these decisions may not always stem from the patient’s choice alone; healthcare providers may also feel pressured to consider euthanasia due to the lack of available resources, leading to ethically fraught situations where the line between patient autonomy and provider-driven decisions becomes blurred [4, 8, 11].

Furthermore, the misconception that palliative care equates to euthanasia can exacerbate this issue. Palliative care aims to enhance the quality of life for patients with severe illnesses through comprehensive support, yet its misinterpretation can lead families to opt for euthanasia instead [23]. This misunderstanding, coupled with the limited availability of quality healthcare, can create an environment where euthanasia is seen as a practical solution rather than a last resort.

### Family resource constraint-motivated euthanasia

The rising costs of healthcare and financial strain on families caring for terminally ill individuals have contributed to discussions about euthanasia as a means of alleviating both financial and emotional burdens [24, 25]. In this study, requests for euthanasia from family members often stem from financial constraints rather than a desire for their loved ones to die peacefully. Families facing substantial medical bills or the inability to afford life-sustaining treatments may prevent euthanasia from escaping overwhelming financial pressures.

Ghana’s cultural values prioritize family unity and support, making open discussions about euthanasia challenging. However, as economic pressures increase and access to modern medical treatments becomes more expensive, families may find themselves in situations where traditional values clash with practical realities. This can lead to difficult decisions that challenge their cultural beliefs about life and death.

The recognition of verbal requests for euthanasia varies widely across countries. In nations where euthanasia is legalized, such as Belgium and the Netherlands, verbal requests are often accepted under strict conditions [26]. However, in Ghana, where euthanasia remains illegal, such requests are not legally recognized, creating further ethical dilemmas for healthcare providers. The lack of explicit legal guidelines complicates discussions around end-of-life care, mainly when families express a desire for euthanasia through neglect or withdrawal of treatment.

The complexities surrounding action-determined euthanasia highlight the need for a nuanced understanding of patient autonomy and cultural values [3]. In Ghana, the legal framework does not explicitly address euthanasia, leading to uncertainties around the ethical implications of such practices. Any discussion about legalizing euthanasia must balance individual autonomy with the deeply ingrained cultural and religious values that shape Ghanaian society [6, 8, 11]. Public discourse on euthanasia, including family resource constraint-motivated euthanasia, remains limited, necessitating inclusive conversations among medical professionals, ethicists, religious leaders, and community members [27].

### Limitations of the study

This study involved only nurses and midwives. Medical doctors, patients with life-threatening diseases, families, clinical psychologists, and social welfare personnel were not included even though they could have shared their experiences and perspectives on end-of-life care decisions. Despite these limitations, the study provides important evidence that is vital to understanding factors influencing Euthanasia and the need to provide terminally ill patients and their relatives with compassionate and dignified care.

### Implication for nursing

The Nursing and Midwifery Council of Ghana, the Ghana College of Nurses and Midwives, the Ministry of Health-Ghana, and the Ghana Health Service should implement structured training for nurses and midwives on euthanasia laws and their implications. Key components of this training should include:

- Legal Education: Provide comprehensive information about the legal status of euthanasia in Ghana, including potential legal ramifications for healthcare professionals.
- Ethical Decision-Making: Focus on ethical principles guiding end-of-life care, incorporating case studies and role-playing to enhance critical thinking and decision-making skills.
- Cultural Sensitivity: Address cultural beliefs surrounding death and end-of-life care to improve communication and trust with patients and families.
- Palliative Care Education: Emphasize pain management and holistic care principles to alleviate suffering and reduce the perceived need for euthanasia.
- Family Education: Extend educational initiatives to families, highlighting their responsibilities in caring for terminally ill relatives and the implications of neglect.
- Resource Allocation: Advocate for improved healthcare resources to support adequate end-of-life care, including access to pain relief and trained professionals.

Additionally, conducting a quantitative study on healthcare professionals’ and the public’s views on the potential legalization of euthanasia in Ghana will provide valuable insights to inform policy.

## Conclusion

The economic implications of euthanasia are intertwined with ethical and cultural dimensions. In Ghana, where preserving life is paramount, the interplay between economic constraints and cultural beliefs can significantly influence public attitudes toward euthanasia. Addressing these complexities is essential for developing informed policies and practices that respect both patient autonomy and cultural values within the healthcare system in Ghana.

## Electronic supplementary material

Below is the link to the electronic supplementary material.

Supplementary Material 1

## Data Availability

The data from which this manuscript was produced is with the corresponding author. It is available on request.
