# Supplementary material for: Navigating end-of-life care dilemmas: a qualitative inquiry of nurses’ and midwives’ knowledge of euthanasia and circumstantial factors influencing euthanasia in a resource-constraint setting
Source: BMC Nurs. 2024 Nov 26;23:859. doi: 10.1186/s12912-024-02527-2 (PMC11590281; doi:10.1186/s12912-024-02527-2)
Supplement: Supplementary file 1 — Supplementary Material 1 [file 12912_2024_2527_MOESM1_ESM.docx]

Data Collection Instruments (Interview Guide)

Appendix B: DEMOGRAPHIC INFORMATION

1. Age (years)……………………………….

2. Place of work………………………………………….

3. Facility ………………………………………………………….

4. Tribe…………………………………………………………………

5. Marital Status…………………………………………………………

6. Profession……………………………………………………………….

7. Specialisation…………………………………………………………..

8. Level of education……………………………………………………….

9. Languages spoken………………………………………………………..

10. Religion…………………………………………………………………..

11. Years of service …..................................................................................

Appendix C: GUIDING QUESTIONS

1. Please tell me what you know about Euthanasia.

Probe

• Source of information

• During training

• During practice

• On the internet

2. Please share with me the types of Euthanasia you know of.

Probe:

• Active

• Passive

• Indirect

• Indirect

• Involuntary

3. Please tell me what the laws in Ghana say about euthanasia

Criminal

Legalized

6. Can you please share with me your thoughts on Euthanasia in health facilities?

Probe

• Good to practice. Why?

• Bad. Why?

7. If the law of Ghana is reviewed to Legalize Euthanasia, what roles will you play to help implement it?

Assist physicians

Administer lethal doses

8. Please share with me the type of Euthanasia you have seen being carried out of practice in any heath facility you have worked in before.

Probe

• In the community

• At the hospital

• Others….

9. Kindly share with me some examples of the methods used so far.

• Drug overdose

• Withdrawal of treatment

• Any other?

10. What more would you like to tell me?

Thank you.
